# Phase I Study to Assess Safety of Laser-Assisted Topical Administration of an Anti-TNF Biologic in Patients With Chronic Plaque-Type Psoriasis

**DOI:** 10.3389/fmed.2021.712511

**Published:** 2021-07-16

**Authors:** Martin Bauer, Edith Lackner, Peter Matzneller, Valentin Al Jalali, Sahra Pajenda, Vincent Ling, Christof Böhler, Werner Braun, Reinhard Braun, Maximilian Boesch, Patrick M. Brunner, Markus Zeitlinger

**Affiliations:** ^1^Department of Clinical Pharmacology, Medical University of Vienna, Vienna, Austria; ^2^Takeda - Pharmaceutical Sciences, Materials and Innovation, Cambridge, MA, United States; ^3^Boehler Life Science Advice, Berneck, Switzerland; ^4^Pantec Biosolutions AG, Ruggell, Liechtenstein; ^5^Department of Dermatology, Medical University of Vienna, Vienna, Austria

**Keywords:** plaque-type psoriasis, topical, etanercept (enbrel), biologic active molecule, laser, phase 1 clinical studies, local tolerability, drug delivery

## Abstract

Ablative fractional laser treatment facilitates epidermal drug delivery, which might be an interesting option to increase the topical efficacy of biological drugs in a variety of dermatological diseases. This work aims at investigating safety and tolerability of this new treatment approach in patients with plaque-type psoriasis. Eight patients with plaque-type psoriasis were enrolled in this study. All patients received (i) ablative fractional laser microporation (AFL) of a psoriatic lesion with an Er:YAG laser + etanercept (ETA; Enbrel® solution for injection) (AFL-ETA), (ii) ETA alone on another lesion, and, if feasible, (iii) AFL alone on an additional lesion. Overall, all treatment arms showed a favorable safety profile. AFL-ETA improved the lesion-specific TPSS score by 1.75 vs. baseline, whereas ETA or AFL alone showed a TPSS score improvement of 0.75 points, a difference that was not statistically significant and might be attributable to differences in baseline scores. Topical administration of ETA to psoriatic plaques *via* AFL-generated micropores was generally well-tolerated. No special precautions seem necessary in future studies. Clinical benefit will need assessment in sufficiently powered follow-up studies.

## Introduction

Psoriasis is a chronic remitting-relapsing, inflammatory disease of the skin, affecting about 2% of the general population (1). Chronic plaque-type psoriasis, also known as *psoriasis vulgaris*, is the form most commonly seen. It is characterized by sharply demarcated, thickened lesions (called plaques) in which both the vasculature and the epidermis are involved, as evidenced by erythema and scale formation, respectively (2). Furthermore, psoriatic lesions can cause pain, itching, and local bleeding. These physical discomforts combined with the potential psychological burden of the disease may interfere with everyday life activities and negatively impact an individual's quality of life (3).

During the last few years, biologics have revolutionized the treatment of moderate-to-severe psoriasis patients. However, there is still a lack of treatment options especially for patients with mild, localized disease when they do not sufficiently respond to, or are intolerant to, topical treatments. Detailed knowledge about the pathogenesis of chronic plaque psoriasis and the central role for the TNF/IL-23/TH17 pathway has led to the development of therapies targeting the pathogenic cytokines, including anti-TNFs, anti-p40 (IL-12/IL-23), anti-p19 (IL-23 specific), anti-IL-17A, and anti-IL-17 receptor antibodies (4). Novel topical agents that can efficiently treat limited skin disease would therefore be highly desirable.

Etanercept (Enbrel®), a genetically-engineered fusion protein acting as a soluble decoy receptor, has been approved as a safe and efficacious treatment option for patients with moderate-to-severe plaque psoriasis in the US, Europe, and a number of other countries. Mechanistically, etanercept binds to the pro-inflammatory cytokines TNFα and lymphotoxin-α (LT-α, also known as TNF-β), thereby neutralizing their biological activity. Etanercept thus mimics the inhibitory effects of naturally occurring soluble TNF receptors, while offering a greatly extended half-life in circulation which allows superior therapeutic activity (5, 6). Due to the rather large size of this molecule (934 amino acids and an apparent molecular weight of 150 kDa), the approved route of administration is subcutaneous injection. Nevertheless, previous studies have shown that also topical administration of TNF blockers might have efficacy in psoriasis (7). However, epidermal uptake of biological drugs is naturally limited by the *stratum corneum*, which functions as the main physical barrier for size exclusion in human skin. Pre-treatment of the skin with fractional lasers increases topical drug uptake, while fractional radiofrequency does not (8). The use of an Er:YAG laser device, with a wavelength that is highly absorbed by H_2_O and therefore requires minimal energy input, results in the creation of a series of micropores with minimal coagulation (9). These micropores permit even large molecules such as biologics to efficiently cross the *stratum corneum* and penetrate into deeper skin layers (10). In a preclinical study, it has recently been shown that etanercept can be delivered efficiently into intact porcine skin at depths ranging from 40 microns to 225 microns. The effect of laser parameters was studied with the goal to optimize clinical delivery rates (11).

In view of the potential synergy between laser microporation and topical etanercept administration, we performed a phase I clinical trial to assess safety and efficacy of ablative fractional laser microporation and topical occlusive application of etanercept in patients with chronic plaque-type psoriasis.

## Patients, Materials, and Methods

This partially observer-blinded, lesion-randomized, intra-patient controlled, 3-arm, monocentric phase I study to assess safety and efficacy of a localized, laser-assisted topical administration of etanercept in patients with plaque-type psoriasis was conducted over 1 year between January 2019 and January 2020.

### Ethics Statement

The study was performed in compliance with the Declaration of Helsinki and the International Conference on Harmonization Good Clinical Practice Guidelines and its amendments. The study was registered under EudraCT no. 2018-001093-19 and EUDAMED no. CIV-AT-20-06-033310, and approved by the local ethics committee and competent authority. All study participants received oral and written information about the study and provided their written informed consent before study enrolment.

Lesions were selected based on similar characteristics, size, and similar location. Treatment was randomly assigned to the respective lesion areas on the first day of treatment. The treatment procedures (etanercept as well as laser) were repeated twice weekly over 6 weeks on the respective lesions. All patients received (i) ablative fractional laser microporation (AFL) of psoriatic lesions + etanercept (ETA; Enbrel® 25 or 50 mg solution for injection in pre-filled pen, marketing authorization holder for Europe: Pfizer Europe) and (ii) ETA alone on another lesion. Four out of eight participants additionally received (iii) AFL microporation alone to treat another lesion (this was only applicable if three comparable lesions could be randomized).

The Er:YAG laser P.L.E.A.S.E.® Professional (Pantec Biosolutions AG, Ruggell, Liechtenstein), with a wavelength of 2'940 nm, a repetition rate of 100 Hz and a pulse length of 225 μs, was used to generate micropores in a 4 or 8 cm^2^ area of a designated plaque. Etanercept (50 mg) solution at a dose of 30 μl/4 cm^2^ or 60 μl/8 cm^2^ was applied to the previously microporated or native surface of the plaque. The treated areas were covered with a transparent dressing for 4 h (occlusion). Patients were asked to document local reactions, adverse events and co-medications in a patient diary. After the screening period, the use of concomitant treatment for psoriasis in all body regions (excluding the three randomized lesions) was restricted to emollients (not supplied), with no pharmacologically active ingredients such as lactic acid, salicylic acid, urea, α-hydroxy acids or fruit acids allowed.

### Patients

Eligible patients were aged ≥ 18 years with chronic plaque-type psoriasis diagnosed at least 6 months prior to baseline who were candidates for topical therapy or phototherapy with at least 2 lesions. Main exclusion criteria were other forms of psoriasis, drug-induced psoriasis, ongoing use of topical corticosteroids, other topical treatments or phototherapy involving study treatment areas and any biological medicinal product (for full in- and exclusion criteria see the above-indicated registries).

### Assessments

Safety assessments included the continuous assessment of the incidence and severity of adverse events (AE), Administration Site Reactions (ASR, defined as itching, redness, swelling, pain, or ulceration), Adverse Device Effects (ADE), local tolerability at the treatment area, laboratory values (blood chemistry, hematology, and lipid panels), monthly pregnancy tests for females of child-bearing potential, and electrocardiograms (ECG) and vital signs.

Assessment of treatment efficacy was based on the established Target Plaque Severity Score (TPSS). To this end, the target plaque was assessed separately for induration, scaling and erythema using a five-point severity scale (0, none; 1, slight; 2, moderate; 3, marked; 4, very marked), and the scores were summed up to yield the TPSS sum score [13-point scale = 0 (no severity), 12 (high severity)]. Assessments were done before the treatment on day 1 (baseline), as well as on days 4, 8, and 13.

### Objectives

Treatment safety as assessed by ASR and AE/ADE was the primary study objective. Treatment efficacy as assessed by TPSS evolution served as the secondary study outcome.

### Randomization and Statistics

Treatment was randomly assigned on the first day of treatment to eligible psoriatic lesions.

The sample size of this study (*n* = 8) was based on clinical and practical considerations rather than formal power calculations. The primary efficacy variable was the TPSS. Changes from baseline (V1) until the last observation (V13) in the TPSS were described and compared between AFL + ETA and ETA only with Wilcoxon's signed rank test in an exploratory manner for the intention-to-treat population. A two-sided significance level of 0.05 was considered for all statistical tests.

## Results

Eight participants (4 females) with a mean age of 43 ± 14 years and a baseline TPSS of 6.9 ± 2 (range 4-10) were included into the study. Detailed patient characteristics are given in Table 1.

**Table 1:** Patient characteristics at baseline.

Participants (female), *n* (%)	8 (50)
Age (years), mean (SD)	43 (14)
Range	23–67
Race, *n* (%)	
Caucasian	8 (100.0)
Other	0
Weight (kg), mean (SD)	89 (37)
Range	55–177
Duration of psoriasis since first diagnosis (years), mean (range)	8 (0.6–19)
Fitzpatrick Score, mean (SD)	3 (1)
TPSS, mean (SD)	6.9 (2.0)
Range	4.0–10.0
BSA (%), mean (SD)	13.7 (6.6)
Range	1.5–23.0

*BSA, body surface area; TPSS, Target Plaque Severity Score; SD, standard deviation*.

### Safety Results

#### Adverse Site Reactions

A total of 64 ASR, all of mild (*n* = 53) or moderate (*n* = 11) severity, were documented in the study. 32 ASR occurred in areas treated with microporation (AFL) and etanercept (ETA). 14 ASR occurred in areas treated with ETA only and 18 ASR occurred in areas treated with AFL only (Table 2). No ASR was graded as severe. Descriptive analysis showed increased ASR—of mostly mild severity—when areas were treated with AFL+ETA as compared to ETA only.

**Table 2:** Adverse site reactions.

**Treatment**	**Adverse site reaction type**				
	**Itching**	**Redness**	**Pain**	**Ulceration**	**Total**
**AFL** **+** **ETA (*****n*** **=** **8)**	8 (25.00)	17 (53.13)	3 (9.38)	4 (12.50)	32
**ETA only (*****n*** **=** **8)**	4 (28.57)	8 (57.14)	1 (7.14)	1 (7.14)	14
**AFL only (*****n*** **=** **4)**	8 (44.44)	9 (50.00)	1 (5.56)	0 (0.00)	18
**Total**	20	34	5	5	64

*Frequency of adverse site reactions (ASR). The percentage is given in brackets*.

#### Adverse Events

A total of eleven AE of mild or moderate severity were documented for five out of the eight study participants, of which most were classified as unrelated to the study procedures: influenza, contact dermatitis on the neck, gastrointestinal bleeding, abdominal cramps (twice in the same subject), headache, constipation, arterial hypertension, hyperlipidaemia, bleeding at laser application site, common cold (two subjects). Furthermore, one serious AE (hospitalization due to arterial hypertension) was recorded and classified as unrelated to the study procedures. No ADE was observed. In addition, one subject experienced two episodes of bleeding at the AFL only laser application site (classified as moderate ASR). No clinically significant deviations in lab results were observed.

### Secondary Objective (Efficacy)

**Figure 1:**
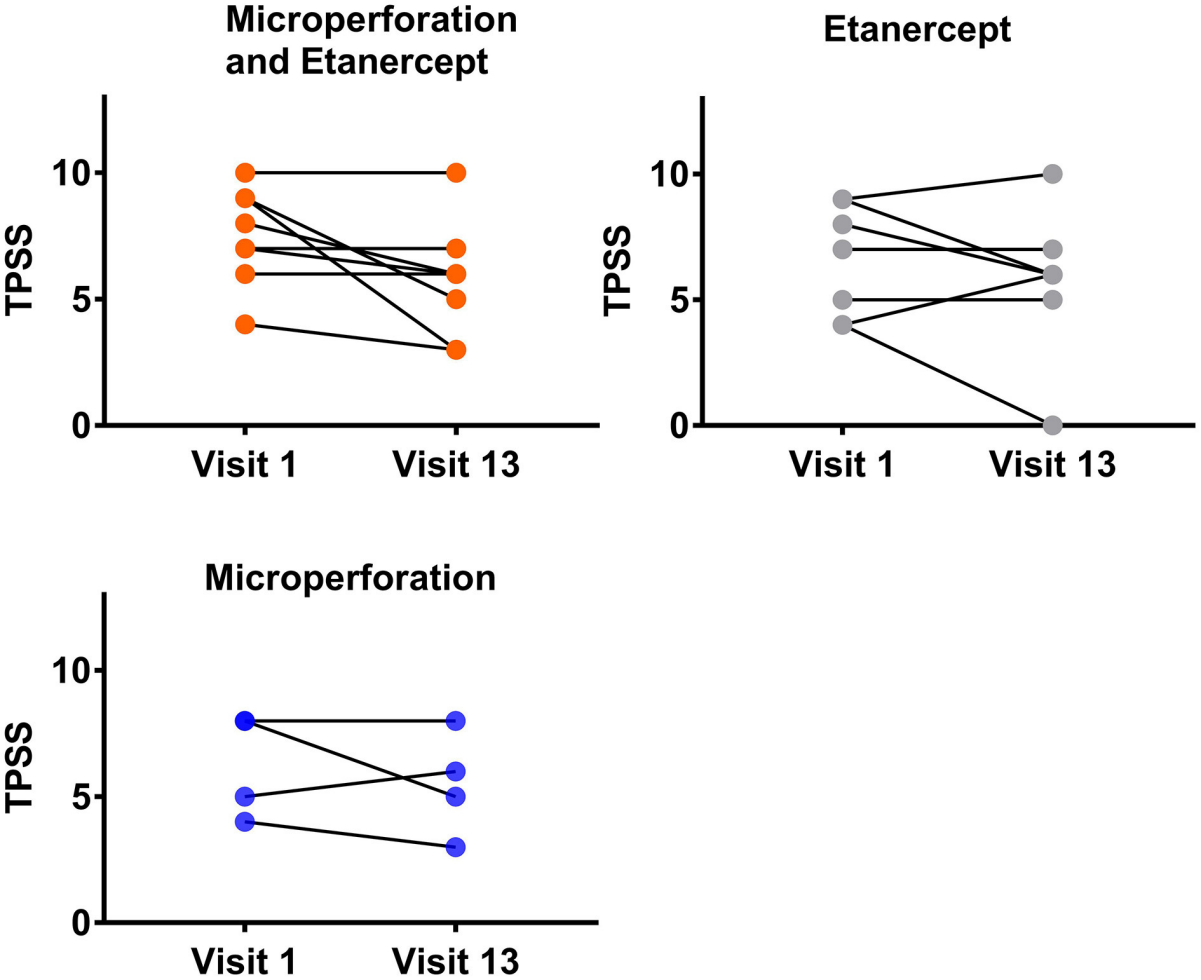
TPSS values (mean ± SD). TPSS was assessed before the respective twice weekly treatments [Er:YAG laser microporation, etanercept (ETA) or combination] over the 6 week study period.

The evolution of the TPSS for the respective treatment over the study period is given in Figure 1.

**Figure 2:**
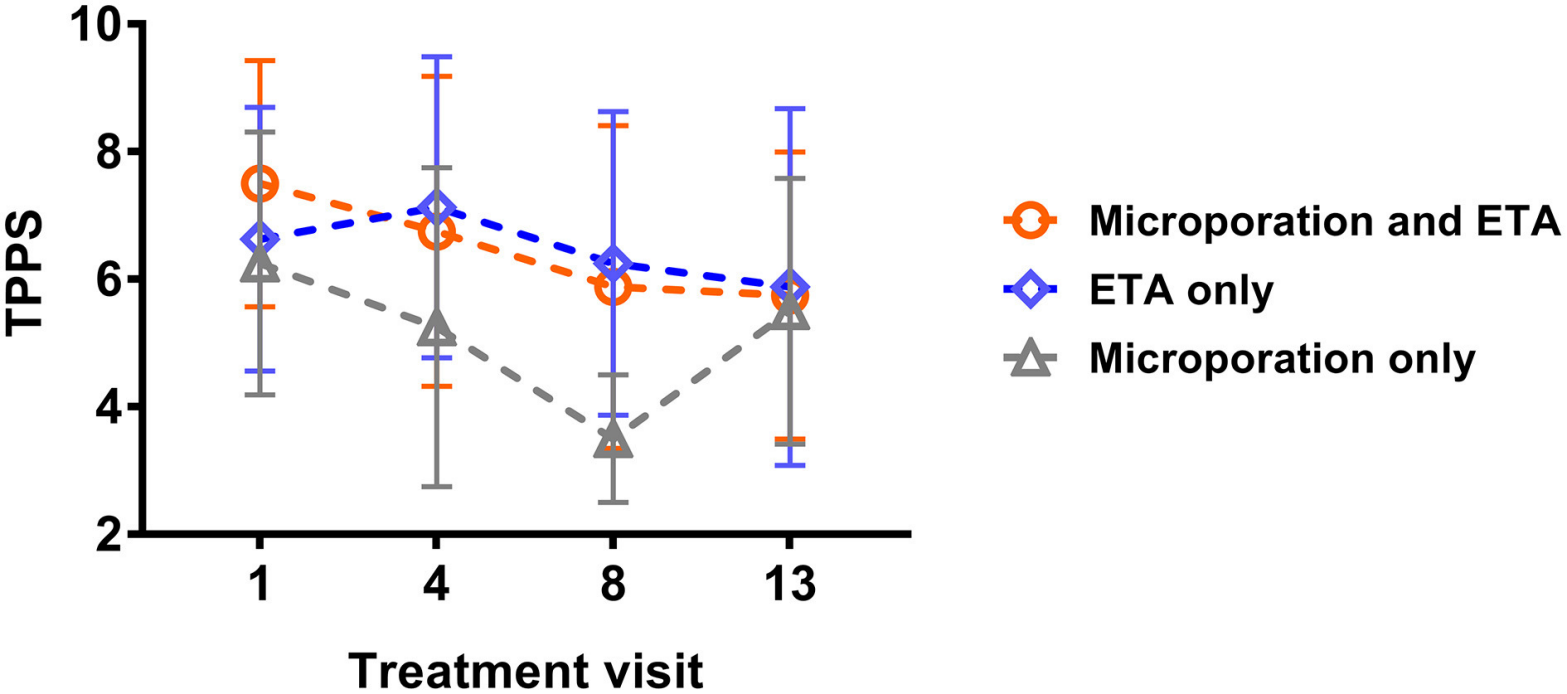
Individual TPSS values at visit 1 and visit 13 for the respective treatment areas. TPSS for etanercept (*n* = 8), etanecept + Er:YAG laser microporation (*n* = 8) and Er:YAG laser microporation (*n* = 4).

Efficacy analysis showed no significant differences between the treatments AFL + ETA and ETA only. However, five patients (62.5%) had higher V1-minus-V13 differences under AFL + ETA than under ETA only, two patients (25%) had the same changes over time in both treatments and only one patient (12.5%) showed a higher difference under ETA than under AFL + ETA (Figure 2). Changes from V1 to V13 under AFL + ETA were not significantly different to changes from V1 to V13 under ETA only (*p* = 0.2813; Wilcoxon's signed rank test).

The raw data from TPSS Total-Score by visit and treatment are listed in Supplementary Table 1.

Plaque lesions selected for treatment of a representative subject are displayed in Supplementary Figure 1.

## Discussion

Results from this study suggest that laser-assisted epidermal delivery of ETA to psoriasis lesions is generally safe and well-tolerated. A comprehensive assessment of risks and benefits associated with either treatment arm (AFL + ETA, ETA only, AFL only) is naturally hampered by the low sample size of a phase I study.

A total of 64 ASR were documented throughout the study. In areas treated with the combination of AFL and ETA 32 ASR, thereof mainly redness (*n* = 14), occurred. By contrast, in areas treated with ETA only 14 ASR and AFL only 18 ASR occurred. Most ASR were graded as mild, none as severe. This leads to the conclusion that topical administration of ETA to psoriatic plaques via AFL-generated micropores in patients with plaque-type psoriasis is well-tolerated. The incidence of ASR was in line with other studies using the same Er:YAG laser system (12).

A comparison of all three treatment groups showed the mean TPSS Total Score evolution (*n* = 8) from treatment visit 1 (V1) to 13 (V13) as follows: AFL + ETA: V1: 7.5, V13: 5.75; ETA only: V2: 6.63, V13: 5.88; microporation only: V1: 6.25, V13: 5.5. While these data indicate the largest numerical improvement in TPSS for AFL + ETA, the numbers did not reach statistical significance. Of note, in contrast to the single treatment lesions, only lesions receiving the combination treatment did not show worsening of the TPSS over the 6 week treatment period (Figure 1). Overall, a mean difference of 1.75 points on the TPSS is in the magnitude of effect commonly used for approval of psoriasis drugs, even though this might be rooted in different baseline scores, therefore warranting future investigation in larger studies.

The strategy of enhancing drug delivery through skin micropores has recently been extensively used for various applications including vaccination (12), topical delivery of small molecules (13), proteins (9), and living human cells *in vitro* (14). Our pilot data provide a basis for further investigation of the combination of AFL + ETA in larger studies. A numerical trend toward lower TPSS in the AFL + ETA group may indicate clinical benefit and justifies follow-up investigation within the framework of larger clinical trials. General benefits of topical drug administration modalities are (i) the possibility to apply high local doses of the active compound and (ii) the prevention—or reduction—of systemic side effects. The combination of skin micropores and topical application of a biological drug was well-tolerated within this study. Local reactions were observed but generally of mild intensity.

The drug formulation was not optimized and due to high fluidity special attention was needed during topical administration. In our case ETA doses of 30 μl/4 cm^2^ or 60 μl/8 cm^2^ was applied to the treatment area of 4-8 cm^2^ in comparison to 50 mg dose in 1 ml needed for systemic efficacy. The lowered economic burden afforded by localized delivery system has been demonstrated in other medical fields as well, most notably with the case of systemic bevacizumab adapted for local intra-ocular delivery for wet age-related macular degeneration (AMD) (15) allowing affordable treatments for many AMD patients, at a global scale. Further development of laser-based microporation technology, using current electronic components and controls can also reduce the cost of instrumentation. The current device used is large, programmable, and designed for clinical application, but miniaturization engineering can reduce unit size to a lower cost with potential for unsupervised at-home applications. Further development in the field of laser-assisted biologics delivery in dermatology can allow applications that stretch beyond psoriasis and are accessible to patients worldwide.

Based on the favorable safety profile of the here investigated laser-medicinal product combination, no special precautions seem necessary for future studies.

In summary, topical administration of ETA to psoriatic plaques *via* AFL-generated micropores in patients with plaque-type psoriasis was generally safe and well-tolerated. The study presented here demonstrates a medical path for utilizing biologics on a local basis for dermatological conditions. Safety of ETA treatment in this context opens up the opportunity to examine the use of other anti-inflammatory and immunosuppressive biologics for topical administration, especially in settings where systemic exposure to the treatment agent would result in greatly reduced local concentrations at the target lesion.

## Data Availability Statement

The original contributions presented in the study are included in the article/Supplementary Material, further inquiries can be directed to the corresponding author/s.

## Ethics Statement

The studies involving human participants were reviewed and approved by Ethics Committe of the Medical University of Vienna. The patients/participants provided their written informed consent to participate in this study.

## Author Contributions

CB, WB, RB, MBo, and MZ designed the study. MBa, EL, PM, VA, SP, and PB conducted the study. MBa, PB, and MZ discussed the data. MBa, VL, MBo, PB, and MZ wrote the manuscript. MZ supervised the study. All authors agreed on publication of this manuscript.

## Conflict of Interest

VL is employed by Takeda, CB is consultant to Takeda. WB and RB are founders of Pantec Biosolutions AG and MBo serves as an advisor for Pantec Biosolutions AG. The remaining authors declare that the research was conducted in the absence of any commercial or financial relationships that could be construed as a potential conflict of interest.

## Supplementary Material

Supplemental Figure 1 Plaque lesions selected for treatment with the respective TPSS scores at baseline and before the last treatment on visit 13 (AFL, ablative fractional laser microporation; ETA, etanercept). Each treated area is about 4 cm^2^.
