# Supplementary material for: Phase I Study to Assess Safety of Laser-Assisted Topical Administration of an Anti-TNF Biologic in Patients With Chronic Plaque-Type Psoriasis
Source: Front Med (Lausanne). 2021 Jul 16;8:712511. doi: 10.3389/fmed.2021.712511 (PMC8322842; doi:10.3389/fmed.2021.712511)
Supplement: Supplementary file 1 [file Table_1.DOCX]

**Supplemental Table 1: Raw data from TPSS total score by visit and treatment**

| **Treatment** | **Patient Number** | **Plaque** | **Visit 1** | **Visit 4** | **Visit 8** | **Visit 13** | **V1 minus V4** | **V1 minus V8** | **V1 minus V13** |
| --- | --- | --- | --- | --- | --- | --- | --- | --- | --- |
| **Etanercept only** | 1 | B | 4 | 4 | 4 | 6 | 0 | 0 | -2 |
|  | 2 | A | 7 | 9 | 9 | 7 | -2 | -2 | 0 |
|  | 3 | A | 4 | 5 | 3 | 0 | -1 | 1 | 4 |
|  | 4 | B | 8 | 8 | 6 | 6 | 0 | 2 | 2 |
|  | 5 | C | 7 | 8 | 6 | 7 | -1 | 1 | 0 |
|  | 6 | A | 9 | 11 | 10 | 10 | -2 | -1 | -1 |
|  | 7 | A | 5 | 5 | 7 | 5 | 0 | -2 | 0 |
|  | 8 | B | 9 | 7 | 5 | 6 | 2 | 4 | 3 |
|  | ***Mean*** |  | ***6.625*** | ***7.125*** | ***6.25*** | ***5.875*** | ***-0.5*** | ***0.375*** | ***0.75*** |
| **Microporation only** | 1 | C | 5 | 4 | 3 | 6 | 1 | 2 | -1 |
|  | 3 | C | 4 | 4 | 3 | 3 | 0 | 1 | 1 |
|  | 4 | C | 8 | 9 | 5 | 5 | -1 | 3 | 3 |
|  | 5 | A | 8 | 4 | 3 | 8 | 4 | 5 | 0 |
|  | ***Mean*** |  | ***6.25*** | ***5.25*** | ***3.5*** | ***5.5*** | ***1*** | ***2.75*** | ***0.75*** |
| **Microporation and Etanercept** | 1 | A | 6 | 4 | 4 | 6 | 2 | 2 | 0 |
|  | 2 | B | 7 | 9 | 9 | 7 | -2 | -2 | 0 |
|  | 3 | B | 4 | 4 | 3 | 3 | 0 | 1 | 1 |
|  | 4 | A | 8 | 7 | 5 | 6 | 1 | 3 | 2 |
|  | 5 | B | 9 | 7 | 5 | 5 | 2 | 4 | 4 |
|  | 6 | B | 10 | 11 | 10 | 10 | -1 | 0 | 0 |
|  | 7 | B | 7 | 5 | 7 | 6 | 2 | 0 | 1 |
|  | 8 | A | 9 | 7 | 4 | 3 | 2 | 5 | 6 |
|  | ***Mean*** |  | ***7.5*** | ***6.75*** | ***5.875*** | ***5.75*** | ***0.75*** | ***1.625*** | ***1.75*** |
