# Supplementary figures and images for: Phase I Study to Assess Safety of Laser-Assisted Topical Administration of an Anti-TNF Biologic in Patients With Chronic Plaque-Type Psoriasis
Source: Front Med (Lausanne). 2021 Jul 16;8:712511. doi: 10.3389/fmed.2021.712511 (PMC8322842; doi:10.3389/fmed.2021.712511)

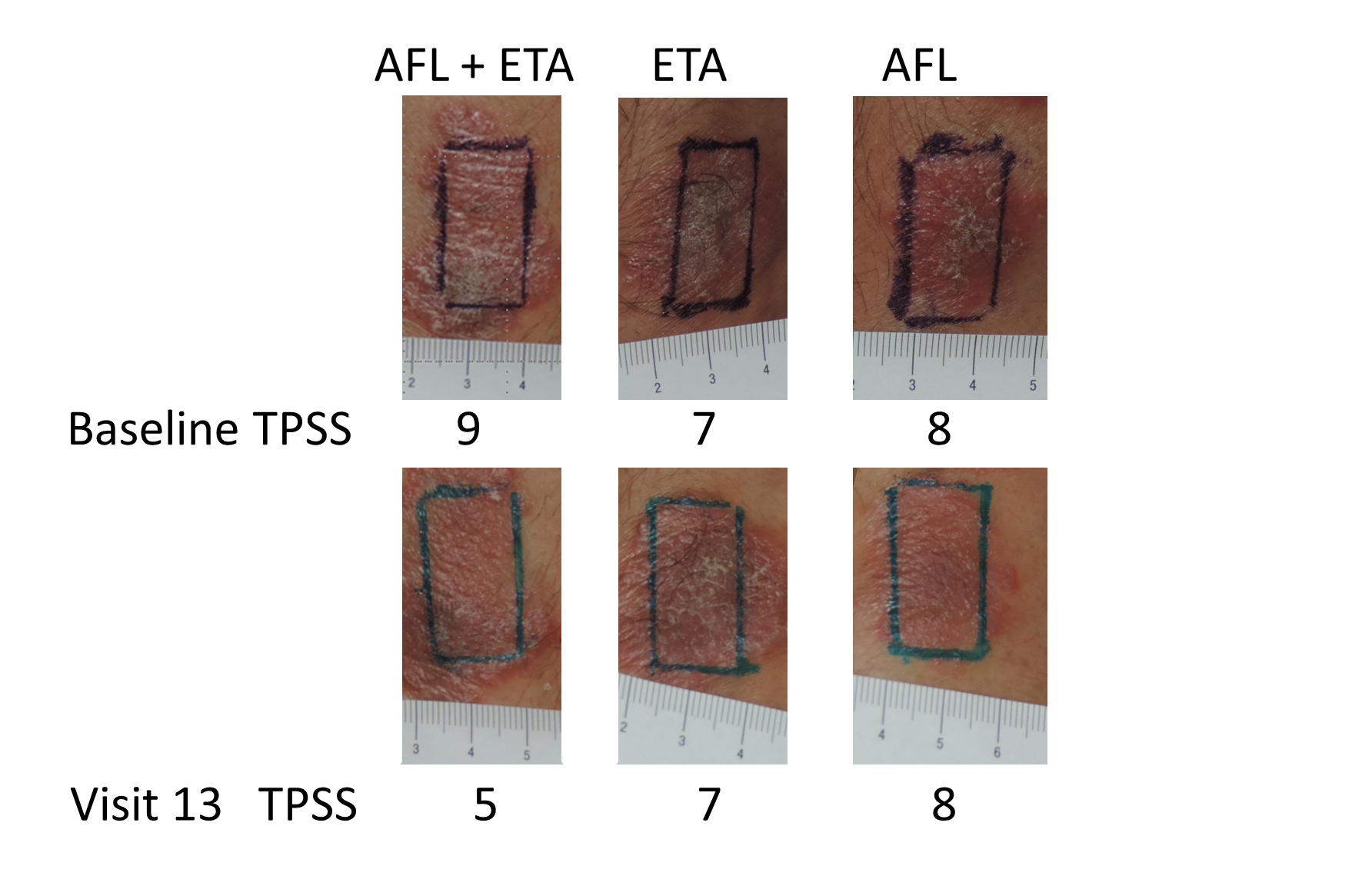

Supplement: Supplemental Figure 1 — Plaque lesions selected for treatment with the respective TPSS scores at baseline and before the last treatment on visit 13 (AFL, ablative fractional laser microporation; ETA, etanercept). Each treated area is about 4 cm2. [file Image_1.TIF]
